# Ammonia reduces intracellular ADMA level in cultured astrocytes and endothelial cells: possible involvement of increased y+LAT2-mediated efflux

**DOI:** 10.1186/2193-1801-4-S1-P28

**Published:** 2015-06-12

**Authors:** Krzysztof Milewski, Jan Albrecht, Wojciech Hilgier, Magdalena Zielińska

**Affiliations:** Department of Neurotoxicology, Mossakowski Medical Research Centre, Poland

**Keywords:** hepatic encephalopathy, hyperammonemia, methylated arginine

Toxic effects of ammonia in the brain are partly related to impaired NO production which depending on the dose/time of ammonia exposure, may be either increased or decrease. Asymmetric dimethylarginine (ADMA), is endogenous NOSs inhibitor and symmetric dimethylarginine (SDMA) is arginine (Arg) transport inhibitor (Teerlink et al., 2009). Previously we reported an increase of ADMA and SDMA concentration in brain of rats with acute liver failure (Milewski et al., 2014), but distribution of the ADMA/SDMA surplus between the particular intra and extracellular compartments has not been studied. Here, we measured the intracellular concentration of ADMA, SDMA and ADMA/SDMA/NO precursor Arg, in cultured cortical astrocytes and rat brain endothelium cells (RBE-4) treated or not with ammonia. In RBE-4 cells not treated with ammonia the ADMA concentration was twice higher and the Arg/ADMA ratio was much lower than in astrocytes, confirming the well documented role of ADMA in endothelial NOS inhibition (Pope et al., 2009). Treatment for 48 h with 5 mM ammonia led to an almost 50% reduction of ADMA and SDMA concentration in both cell type. Since ammonia-dependent Arg transport in astrocytes is specifically mediated by the heteromeric Arg/Gln transporter y+LAT2 (Zielińska et al., 2012), we speculated that this may also hold for both Arg derivatives. Indeed, silencing of the y+LAT2 gene diminished the reduction of intracellular ADMA concentration caused by ammonia treatment in astrocytes. Moreover, the y+LAT2-dependent component of ammonia-evoked Arg uptake was reduced in the presence of ADMA in the medium. The results suggest that increased ADMA (and possibly SDMA) efflux mediated by upregulated y+LAT2 may be one of the ways in which ammonia interferes with intra-astrocytic ADMA content and, subsequently, NO synthesis. Studies are underway to establish if the same sequence of changes holds for ammonia-treated cerebral endothelial cells.
